# The Sound of a Circular City: Towards a Circularity-Driven Quietness

**DOI:** 10.3390/ijerph191912290

**Published:** 2022-09-27

**Authors:** Aggelos Tsaligopoulos, Stella Sofia Kyvelou, Michalis Chiotinis, Aimilia Karapostoli, Eleftheria E. Klontza, Demetris F. Lekkas, Yiannis G. Matsinos

**Affiliations:** 1 Acoustic Ecology Laboratory, Department of the Environment, University of the Aegean, 81100 Mytilene, Greece; 2 Department of Economic and Regional Development, School of Science of Economics and Public Administration, Panteion University of Social and Political Sciences, 17671 Athens, Greece; 3 Department of Water Resources and Environmental Engineering, School of Civil Engineering, National Technical University of Athens, Heroon Polytechneiou 9, 15780 Zographou, Greece; 4 School of Architectural Engineering, Aristotle University of Thessaloniki, 54124 Thessaloniki, Greece; 5 Waste Management Laboratory, Department of the Environment, University of the Aegean, 81100 Mytilene, Greece

**Keywords:** circular economy, bioeconomy, noise control, soundscape, noise model, noise map, green walls, electric vehicles, quietness

## Abstract

The circular economy paradigm can be beneficial for urban sustainability by eliminating waste and pollution, by circulating products and materials and by regenerating nature. Furthermore, under an urban circular development scheme, environmental noise can be designed out. The current noise control policies and actions, undertaken at a source–medium–receiver level, present a linearity with minimum sustainability co-benefits. A circular approach in noise control strategies and in soundscape design could offer numerous ecologically related co-benefits. The global literature documenting the advantages of the implementation of circular economy in cities has highlighted noise mitigation as a given benefit. Research involving circular economy actions such as urban green infrastructure, green walls, sustainable mobility systems and electro-mobility has acknowledged reduced noise levels as a major circularity outcome. In this research paper, we highlight the necessity of a circularity and bioeconomy approach in noise control. To this end, a preliminary experimental noise modeling study was conducted to showcase the acoustic benefits of green walls and electric vehicles in a medium-sized urban area of a Mediterranean island. The results indicate a noise level reduction at 4 dB(A) when simulating the introduction of urban circular development actions.

## 1. Introduction

Historical evidence underlines that environmental noise has been a problem associated with urban life since the distant past (Juvenal, Satire III. 232–238 And Then There is the Traffic) [1]. The by-product of noise, coupled with economic activities, can be considered as the sound of progress. Noise has always been tied with several aspects of society [2], but it was mainly the industrial revolution that re-shaped the world along with the sound environment of cities [3]. Noise is a form of energy leakage in the urban environment [4] affecting urban residents and the overall environmental quality. Among the numerous quantitative and qualitative ways to interpret the specific acoustic phenomenon [5], noise can also be described as an externality of urban development [6]. The increasing health-related hazards [7,8] caused by this externality have created both the need and the economy to tackle it in order to create a state of urban quietness. There are various scales and methods to achieve noise reduction, with various costs and benefits, functioning as an interrelated system of labor, exchange and consumption [9].

Cities stand as the forefront of a global multifaced change and present the dichotomy of being both the cause of and the solution for global and local climatic crisis phenomena [10,11,12,13]. According to data provided by the World Bank, about 75% of Europe’s population [14,15] and 57% of the world’s population live in urban areas. Cities are hosting the majority of the human population with an increasing trend and are also responsible for the majority of energy consumption and carbon emission [16]. Thus, an adaptable city with the appropriate infrastructure is of high importance [17]. The recent strides made towards urban sustainability [18,19], urban adaptation [20], urban regeneration [21,22] and resource looping have been incorporated into the recently expanded concept of circular economy and urban circular development [4,23,24,25,26].

The main scope of this research is to approach and analyze the symbiotic relationship between urban circular development and urban quietness. This means that actions of urban circular economy can result in the co-benefit of quietness, and at the same time, a truly quiet urban sound environment can be achieved, planned or designed with circularity principles. Moreover, we conducted a preliminary study on the sound environment of a small-scale urban area in an insular Mediterranean city. Apart from documenting the current noise conditions in the area, the aim was to offer an initial perspective on the acoustic benefits that may occur following the city’s transition towards a circular economy model. Recently, the Aegean Bioeconomy project endorsed the sustainable and circular bioeconomy in the insular regions of the North and South Aegean (Greece) through the development of new technologies and knowledge sharing [27]. The worldwide growth of circular economy strategies and experiments, and especially the ones taking place in the Mediterranean, are of paramount importance mainly due to the uniqueness of the natural and cultural characteristics that compose the Mediterranean identity [28,29].

This research is structured around key theoretical concepts and experimental approaches. Firstly, a theoretical assessment of the association of urban circular development and urban quietness is conducted. To this end, a critique of the current one-dimensional noise control methods lacking sustainable co-benefits is highlighted. Furthermore, the circular development actions directly associated with the sound environment of a city are discussed. Specifically, the benefits and drawbacks of circular actions involving green walls and electro-mobility are analyzed. Secondly, the experimental elements of this research encompass a noise measurement protocol in order to highlight the current noise conditions of the case study area, followed by the noise modeling approach regarding the forthcoming implementation of the above circular actions in the city under consideration.

## 2. Background

### 2.1. Circular Economy and Urban Sustainability

Circularity contradicts the “take-make-waste” linear model and encourages a restorative and regenerative economy model, promoting, among other actions, resource looping [30]. Urban circular economy refers to a regenerative system in which resource input and energy leakage are minimized by closing material and energy loops [26] using actions such as repairing, reusing, remanufacturing, refurbishing and recycling [31]. The long-term aim of circular economy is to integrate the cycles of nature into the flows of materials [32]. This is an ideal approach for implementing the basic principles of green growth [33].

A plethora of cities are currently adopting circular economy strategies [34]. Amsterdam, Bristol, London, Peterborough, Paris, Stockholm, New York, several cities of China and other areas are testing and validating circularity at several scales [35,36,37]. Among the most important factors towards an urban circular transition are:

- Resource looping incorporating reuse and recycling systems such as gray water re-purposing;
- Adaptation schemes such as flexible infrastructure, according to the changing needs of a community;
- Ecological regeneration that involves the promotion of natural cycles, thus improving well-being [25];
- Sharing of products and digital tools to facilitate the procedure;
- Optimization of the energy production towards local and renewable methods;
- Upgrading of buildings, e.g., with green walls and green rooftops; and
- Mobility systems that are compatible with a more sustainable way of commuting [4].

Through the incorporation of the above circular actions, several ecological, social and economic co-benefits are expected, including noise level reduction, decreased greenhouse gas emissions, individual well-being, environmental and ecosystem health and job opportunities boosting the local economy [23,27,31].

The circular economy model and the encouragement towards resource looping can minimize waste and lay the groundwork for a truly sustainable urban environment, while setting the standards of a new urban resident lifestyle [38,39]. Either voluntary or involuntary changes in lifestyle may affect the environment [40,41]. Recently, it has been thoroughly documented that a major involuntary alteration in lifestyle resulted in significant environmental changes [42].

During the COVID-19 pandemic, research regarding the assessment of urban sound environments highlighted the major changes—and in some cases, benefits—that resulted from this unfortunate drastic change in lifestyle. The lockdowns applied during the pandemic to minimize the infectivity of COVID-19 altered the sound environments of cities. The reduced road traffic and the overall limited human activities outdoors created less eventful but quieter urban acoustic environments [43,44,45,46,47]. Furthermore, this lifestyle alteration created the need for acoustically healthier building interiors, aiming towards the well-being of tenants [48,49]. Numerus articles describing the positive and negative characteristics of soundscapes highlighted natural sounds as the most preferable sound mark of both interior [48] and exterior sound environments [50,51,52,53]. Due to the direct effects of urban dwellers’ lifestyle on several aspects of the built environment, including energy consumption [54], a new welcoming change is expected that could be the result of a “circular lifestyle”.

Sustainability is the main driving force for the implementation of circular actions. These actions, either institutionally or socially driven, involve a fundamental change in the linear economy model [36]. Concurrently, the transformation in urban inhabitants’ lifestyle may play a pivotal role in the success of this global endeavor. This new sustainable way of urban living, combining new ways of producing, using and recycling with a more sustainable way of transportation and new infrastructure that can promote looping and ecological regeneration, may result in a new urban sound environment. Therefore, the hypothesis that a circular city will be a quiet city is gradually shaped.

### 2.2. The Linearity of Noise Control and the Circular Approach towards Urban Quietness

The decline in the environmental quality in urban areas due to pollution, unsustainable development and unplanned growth is the cause of rapid change in both the landscape and the sound environment of a city [55]. Noise in excessive amounts can cause mental health problems, hearing complications, sleep disorders and even cardiovascular disease [56]. The fact that most of Europe’s population is exposed to road traffic noise levels above those suggested by the World Health Organization’s 55 dB(A) limitation [7] shapes the need to minimize environmental noise and promote urban quietness [57]. A circular approach towards quietness is proposed and an opportunity for sustainability co-benefits is presented.

**Figure 1:**
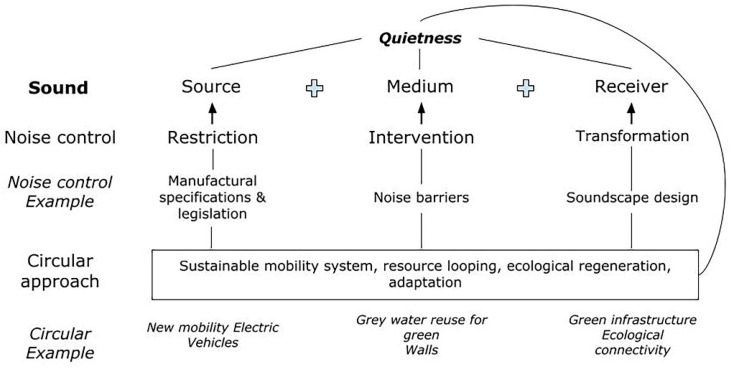
The circular approach to quietness. Noise control actions individually or in combination may result in quietness. Similarly, circular development actions can result in quietness with the co-benefit of urban sustainability.

Noise control strategies usually present a linearity. The pivotal characteristics of sound include a sound source, a medium (gas, liquid, solid) and a receiver. Regardless of the polysemy of noise and the way it is perceived in context, the increased decibel levels represent an important health hazard. As it can be seen in Figure 1, the most common noise control methods introduced individually or in combination to achieve reduced noise levels are (a) either a direct restriction of the noise source, (b) an intervention in the medium between the source and the receiver and (c) a structural or aural transformation at the receiver level [58,59].

Managing noise directly at its source is a common but still complex and expensive process [60]. Road traffic and other types of transportation noise are one of the most common sources of noise that burdens the well-being of urban dwellers [61,62,63]. The management of transportation noise directly at the source is a difficult task due to the different stakeholders involved, similar to vehicle manufacturers [58].

The second level of noise management concerns the interference at the medium level. The most common approach is the installation of noise barriers [64,65,66] usually placed on high-traffic roads in order to mitigate the effects of traffic noise along the highway. They block the direct path of sound between the noise source and the exposed receiver [67].

The methods of noise reduction at the receiver level are determined in the concept of the receiver in each case study. Insulated windows [68] and building façade sound insulation systems [69] can be considered as measures taken at the receiver level. Nevertheless, the notion of a receiver can be broadened and involve the numerous stakeholders present in the built environment, such as a public urban green space. In that sense, a receiver could regard the human health and well-being [70] and the natural capital [71] (e.g., birds) threatened by the unwanted effects of noise. A way to achieve this is the shaping of the built environment in order to design the soundscape and replace unwanted sounds [72,73,74].

A circular approach in urban planning [25] could offer sustainability co-benefits and highlight urban quietness as the carrier of a truly sustainable city. The three essential circular actions in a city are resource looping, ecological regeneration and adaptation [24]. Resource looping refers to the recycling and energy recovery actions, similar to the gray water reuse for green roofs and walls [75,76,77]. Additionally, ecological regeneration and adaptation refer to the design of infrastructure that enables the circular flow of resources, across sectors, within urban systems. Furthermore, since the major contributor to environmental noise and air pollution is urban traffic, a sustainable mobility system is crucial. The use of electric vehicles could provide such a solution, but much work is still needed to integrate this circular activity in a truly sustainable paradigm [78].

**Figure 2:**
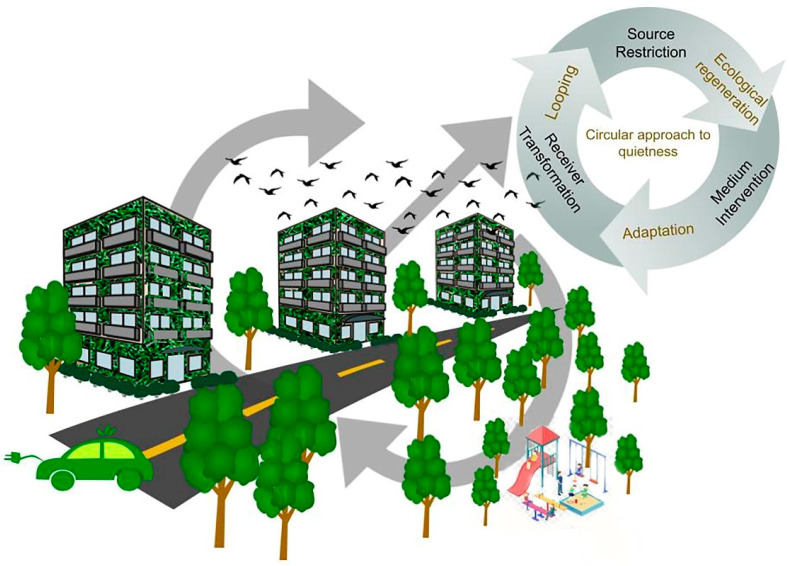
The circular approach to quietness. The noise control actions, either individual or in combination, implemented through a circular approach may result in a truly quiet and sustainable urban environment.

As described in Figure 2, a circular approach in noise control actions, involving a sustainable mobility system such as the use of electric vehicles, resource looping, adaptation and ecological regeneration, could be beneficial for the overall urban environment.

### 2.3. Circular Development Actions That Benefit the Urban Sound Environment

The major components of a circular city that are expected to influence the urban sound environment are modifications of the built environment, urban green infrastructure, mobility and transportation and overall lifestyle alterations.

#### 2.3.1. Urban Green Infrastructure

For improving soundscape quality, natural sounds seem to be preferred and promoted [79,80]. The promotion of natural sound could be supported through the use of renewable biological resources such as the establishment of plants, acting as part of noise mitigation techniques with noise insulation and sound absorption properties [81,82]. Applying the bioeconomic approach [83] in the management of the urban acoustic environment in terms of materials and strategy plans regarding noise mitigation could be a promising field with multiple co-benefits.

Green walls and green roofs can act as small habitats or stepping stones improving the dispersal of species in the urban fabric [84], while playing a critical role in the ecological functionality and connectivity of fragmented areas [85]. In addition, they enhance the dispersal of many species found in urban green areas such as parks and reduce the barrier effects created by urban morphology [86]. The differentiation between green façades and living walls must be clarified. Green façades are created with the use of climbing plants that grow on an existing vertical surface. On the other hand, living walls contain soil that supports growing plants [87,88].

Greening systems, including green walls, living walls and green roofs, provide a series of benefits at city and building scales. Several environmental, economic and social benefits can stem from the introduction of green wall systems [89]. An increase in urban biodiversity at a city scale can be expected [90]. Nevertheless, biodiversity is a complex and multi-level concept expanding from gene level to species richness [91]. The effectiveness on biodiversity appraisal of greening techniques for buildings is associated with the size of installation, its isolation from other green spaces, plant heterogeneity and age [92]. Green walls can improve microclimate and, more specifically, the ventilation capacity in a compact street canyon, leading towards effective accumulation of traffic exhaust pollutants [74]. Additionally, a mitigation of the heat island effect can be achieved [93], substantially improving the climatic conditions of an area and decreasing energy consumption due to cooling. Finally, plentiful social and economic benefits [94] can be expected at city scale. On the other hand, greening systems produce several indoor benefits, mainly involving building energy performance, insulation advantages and indoor thermal improvement [36]. Several disadvantages and risks have been reported [95], including the substantial cost of construction and maintenance, the possibility of future damage to building structure, the appearance of several nuisances caused by miscalculated design [96] and the attraction of pests and disease vectors similar to mosquitoes, especially in rainy seasons.

Towards the effort of reducing the environmental impact of material production and consumption, even in green wall applications, the circular economy paradigm could be applied [97,98]. Furthermore, the concept of circular urban metabolism stands as a holistic, multidimensional way to deal with waste and noise management [99] and promote circularity [100]. In agreement with the desired circularity in cities, green walls were proven useful in several cases as pollutant removals and were tested as a gray water treatment medium [101].

Despite the multidimensionality of the concept of noise regarding its perception and effects, it can still be treated as a sound created by the vibration of a substance [80]. The collision of a sound wave with a material (wall) results in a series of effects, including reflection and absorption. The porosity of the impacting material in which the sound waves enter causes friction between the air molecules and the pores of the material, resulting in absorption [72]. The sound absorption of materials is evaluated through the sound absorption coefficient, which is the ratio of absorbed energy to incident energy and is represented by α. The sound absorption coefficient has a range 0 ≤ α ≤ 1, with α = 1 indicating that the acoustic energy can be absorbed in its entirety [102].

The degree of sound absorption of a material, including vertical gardens, varies among frequency range [103]. For higher frequencies (1600–5000 Hz), the sound absorption properties of green walls are found to be greater and thus more effective [60]. The results from several experiments testing the absorption coefficient of vertical gardens or living walls, which indicated that the sound absorption effects derive mainly from the soil and secondly from the plants [104]. The average of the frequency range is more effective to use, and recent studies presented a variety of sound absorption coefficients related to the material used in the construction of green walls [105,106,107].

#### 2.3.2. Circular Mobility

It has been documented that population growth and the number of vehicles in a city are increasing simultaneously [108]. Moreover, the road infrastructure in terms of quantity, quality and accessibility is not in sync with the population and increasing vehicle rates [109]. In addition to the above urban challenges, the energy crisis, the changing climate and the fact that transportation is the major contributor to greenhouse gas emissions [110] and environmental noise [111] create the need to establish a more sustainable urban mobility system [112].

Electric vehicles (EV), compared to conventional internal combustion engine vehicles (ICEV), enable the reduction in environmental pressures [113] and contribute to a more sustainable urban transport system [78]. Due to the promising benefits deriving from the use of EVs, efforts of environmental life cycle assessments are being conducted [114] in order to optimize the specific product in terms of its sustainable production [115,116], viable use and charging [117], and finally, the end of life, including recycling and re-use options [118,119].

It is undeniable that the specific form of clean transport deals with challenges regarding the degree of sustainability in the beginning and end of its life cycle. The problem regards mainly the increased use of natural recourses, needed for the lithium-ion batteries employed in EVs [120]. The predicted resource challenges, the supply chain risks, the extraction of materials such as lithium and cobalt from vulnerable ecosystems and the social risks, e.g., the child labor [78], are some of the issues that need to be addressed. Consequently, it is safe to say that a circular economy approach is of vital importance from the early stages of EV construction [121]. Low-carbon city electrification and clean transport involving EVs can support the deviation of environmental pressures and strengthen circular urbanism [122].

EVs and electro-mobility in general can be fundamental inputs of the circular city [121] and present several co-benefits, including noise mitigation [123]. In direct comparison to ICEVs, the noise level difference of EVs could be as high as 6 dB(A) [124]. Nevertheless, it has been documented that above 40 km/h, the noise difference between EVs and ICEVs is alleviated due to the fact that the rolling noise propagating from the tire–road friction masks any other type of sound [125,126,127]. Therefore, the soundscape benefits that may derive from the implementation of electro-mobility are subject to the driving behavior of urban dwellers [128]. Furthermore, as discussed previously, noise intensity is not the only means to describe a sound environment. Other descriptors such as acoustic complexity are equally important. The latter can be achieved through the use of green walls considered as a passive acoustic insulation system for buildings [129]. According to the global literature, it can be assumed that the minimization of ICEVs will release a wide frequency range and liberate an occupied part of the urban sound palimpsest, thus allowing other sounds to be heard [123,130].

## 3. Materials and Methods

### 3.1. Case Study Area

Mytilene is a medium-sized Mediterranean city located on the island of Lesbos (North Aegean, Greece). Islands of the Mediterranean similar to Lesbos are notable for their biological endemism. They are encapsulating various biological and anthropological processes within their borders and exhibit a unique soundscape as part of their natural and cultural heritage [131].

Similarly with other medium-sized cities in Greece, Mytilene embodies the Mediterranean urban identity, including specific natural and cultural heritage characteristics [132]. Nevertheless, Mediterranean cities, including Mytilene, have a fast urbanization rate and unfortunately showcase, in principle, a lack of a sustainable urban development [133].

**Figure 3:**
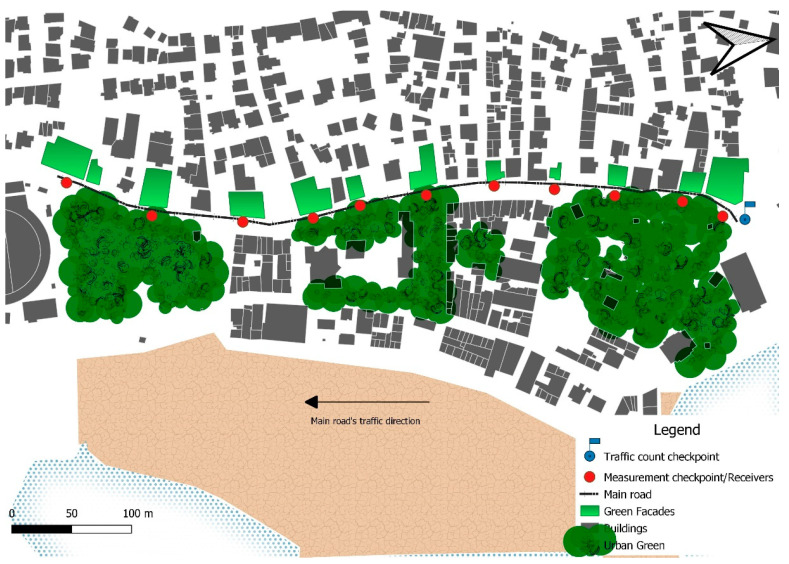
The small-scale urban area located in the city of Mytilene. Point 1 represents Karapanagioti Park and point 2 is Agias Eirinis Park. The red dots denote the measurement checkpoints.

For this study, a small, urbanized area located in the city center of Mytilene was assessed (Figure 3). In the city of Mytilene, as in other cities with a waterfront, the road traffic noise emanating from the adjacent streets deteriorates the quality of a sound environment that could offer, alternatively, a restorative and quiet urban environment [134]. The case study area includes two highly visited urban green spaces and part of a heavily used local street connecting, among other destinations, the city’s main harbor with the island’s airport. Furthermore, the area is of mixed usage, standing as both a dense residential area and a commercial area including shopping centers, offices and other businesses.

The two urban green spaces included in the case study area are (a) the Agias Eirinis Park, which is approximately 12,000 m^2^ and mainly consists of false acacias (*Robinia pseudoacacia*) and other deciduous trees and shrubs, and (b) the Karapanagiotis Park located next to the city’s local soccer stadium, which is approximately 9500 m^2^, and consists of mulberries (*Morus alba*), false acacias (*Robinia pseudoacacia*) and other tree species and large shrubs similar to bay laurels (*Laurus nobilis*). In both green spaces, numerous bird species can be identified with great seasonal variations, including hooded crows (*Corvus cornix*), common blackbirds (*Turdus merula*), great tits (*Parus major*), common chiffchaffs (*Phylloscopus collybita*), European robins (*Erithacus rubecula*), Eurasian blue tits (*Cyanistes caeruleus*) and white wagtails (*Motacilla alba*) [135].

The structural characteristics of the area include relatively new buildings of high altitude with mixed façades of glass and concrete. The road surface is a flat, smooth asphalt without potholes or any other visual surface defects.

The case study area is considered of high importance for one more reason—a bioeconomy and circular economy initiative is taking place in the city of Mytilene, in the framework of a broader relevant policy addressing the insular regions of the North and South Aegean. Future plans regarding resource looping, green walls and roofs, ecological connectivity plans and other similar circular and sustainable activities are being shaped.

### 3.2. Environmental Noise Assessment, Noise Modeling and Receiver Noise Exposure Mapping Techniques

To assess the noise conditions of the specific case study area, the guidelines provided by the CNOSSOS-EU road traffic noise model were implemented [127,136].

The CNOSSOS-EU noise model using the CadnaA noise prediction and noise mapping software (CadnaA—State-of-the-Art Noise Prediction Software, Version 2021 MR1|DataKustik GmbH, Gilching, Germany) considers the digitized roads as sources of noise and the digitized urban structures (buildings) as obstacles for the noise interpolation to take place. Regarding noise from roads, real-time noise level measurements can be interpolated into a noise map or modeled through traffic flow composition.

The reference conditions used in the specific road traffic noise model are the following:

- Constant vehicle speeds;
- Flat roads;
- Air temperature τ ref = 20 °C;
- Dry road surfaces; and
- No studded tires.

The data regarding the equivalent continuous sound level (L_eq_, dBA), along with the simultaneous traffic counts, were collected for a 10-day period and later averaged, during the summer of 2022 (25 July 2022–10 August 2022), avoiding weekends.

The road traffic noise was simulated through traffic flow composition. Since roads in an agglomeration are considered a predominant noise source, the number of vehicles per hour and per category (light vehicles, medium-heavy vehicles, heavy vehicles, mopeds and motorcycles) for each road is required. As it can be seen in Figure 3, a traffic count check point was selected. Due to the morphology of the road network, the specific point served as a panopticon, thus enabling the simultaneous documentation of the passing vehicles. The counting and classification procedure was uncomplicated because the specific road has one direction and two lanes of traffic and was manually conducted simultaneously with the noise level measurements. Finally, the resulted counts for each type of vehicle for the 10-day period were averaged in order to be used as input information for the noise model. The day-by-day traffic count results are being reported below.

Topographical data for the case study area were gathered. More specifically, information regarding the following urban morphology characteristics were collected and later visualized (Figure 3) in QGiS v. 3.22.1 Białowieza (QGIS Development team 2021, Johannesburg, South Africa):

- Detailed cartographic representation of the area under consideration (buildings, roads, vegetation).
- Building height and location: In this case, the highest building was 20 m high.
- Façade material type: In this case, the surrounding buildings’ façades consist of a mixture of concrete and glass materials.
- Vegetation height and location.
- Road type classification (motorway, ordinary road, local).
- Road surface type: In this case, it was asphalt concrete.
- Traffic light location and operation: In this case, 4 traffic lights that do not operate during the night period.
- Vehicle speed: In this case, 50 km/h for passenger vehicles and motorcycles and 40 km/h for trucks, according to the Greek legislation.

For validating the simulated results, successive noise level measurements were conducted at 11 noise monitoring checkpoints. The checkpoints had a distance of 50 m from each other, and each 10 min measurement was conducted at a height of 1.5 m above ground level during the day period (9:00–10:00 a.m.).

The noise level measurements were conducted with the sound level meter pointed at the noise source (road traffic) with an angle of incidence of 0° with respect to its main axis.

The 01dB Fusion class 1 smart noise monitor with the GRAS 40AE free field microphone were used to collect noise data, with 51.2 kHz sampling frequency and a 1 s logging interval. The device was calibrated before data collection using a calibrator, as required for all Class 1 measuring instruments and in accordance with the specifications of EN61326-1:1997+A1:1998 [137]. The software dBTrait v. 6.3.0 (ACOEM, Limonest, France) was used to process the environmental noise data collected.

The model’s output was a receiver noise exposure map visualizing the propagation of road traffic noise deriving from the vehicle composition of the road under consideration.

### 3.3. A Methodological Approach towards a Circular Acoustic Urban Environment Assessment—Modifying the Absorption Coefficient of Façades and Correcting Electric Vehicle Propulsion Noise

In order to simulate the effect of green walls on the sound environment of the case study area and plan for future circular “safe to fail” projects, the absorption coefficient of the buildings was increased to fit the guidelines provided by the literature.

A key factor that determines the sound absorption properties of a green wall system is the porosity of the material and its layer thickness. A recent study portrayed the sound absorption coefficients for various frequencies. Lower frequencies (100–315 Hz) present a range of sound absorption coefficient of 0.59–0.80, and mid-frequencies (400–1250 Hz) and high frequencies (1600–5000 Hz) present a coefficient of 1.00. Finally, a recent evaluation of green walls as a passive acoustic insulation system for buildings resulted in an absorption coefficient of 0.40 [129].

For this research, the sound absorption coefficient of the façades was increased to a degree of α = 0.40 for the 1/3 octave spectrum according to the guidelines provided by the German standard RLS-90 [138,139].

Specifically, as presented in Figure 3, the absorption coefficient of building façades directly facing the 11 checkpoints was increased.

To simulate the introduction of EVs to the road network of the area, correction coefficients on the noise levels of the light vehicles counted were implemented. The CNOSSOS-EU noise model, apart from being the most flexible among other noise models, offers an open vehicle category in order for EVs to be included [125,127].

Since EVs are considered quieter vehicles, the noise emission measurements conducted in environments that include background noise is difficult, especially concerning several frequencies. Therefore, as presented in Table 1, the EV noise reduction coefficients were considered for a narrower frequency range, and more specifically for the octave 125 Hz to 4000 Hz [125].

For this research, the correction coefficients for the propulsion noise of EVs were implemented for the light vehicles that were counted (Table 1). Due to the lack of experimental data that could provide EV correction coefficients for heavier vehicles and two-wheelers, we decided to keep these cases unaltered. Therefore, the ICE light vehicles, which in this case were dominant in relation to other vehicle categories, were substituted with simulated EVs.

For the specific small-scale research, we determined the statistical differences of the resulted measured and simulated noise levels (L_eq_ dB(A)) using the SPSS software (IBM SPSS Statistics, Version 28.0.1.0, IBM, Armonk, NY, USA). Descriptive statistics were used in order to illustrate the mean, median, standard deviation, skewness and kurtosis of the acoustic indicators. Secondly, an appropriate mean difference comparison test was chosen according to the distribution of the data. The null hypothesis H_0_ was that there is no significant difference in noise level conditions before and after the implementation of circular development actions. In order to estimate the magnitude of difference between the two means of indicators, the effect size for t-tests was estimated [140,141]. More specifically, the effect size according to Cohen’s d classification of effect sizes was calculated by dividing the paired samples’ mean difference by the pooled estimated standard deviation.

## 4. Results

**Figure 4:**
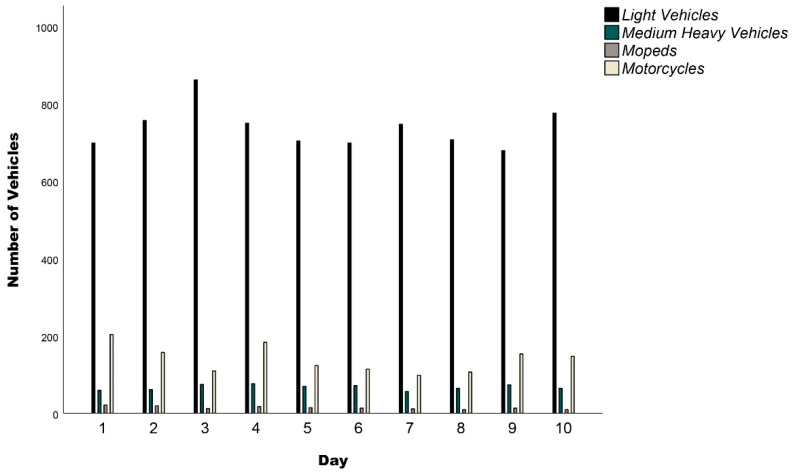
The traffic count data per vehicle category for the 10-day sampling period.

The traffic counts conducted resulted in a fleet that is presented in Figure 4. A total of 10,584 vehicles (M = 690) were counted during the 10-day sampling period. As expected, the light vehicle category presented the highest quantity of all vehicles, reaching 76% of the total vehicles. Unsurprisingly, mopeds where the second most used vehicle (14.3%), which is typical for a Mediterranean island. The remaining vehicle categories presented the lowest percentages among the counted vehicles.

**Figure 5:**
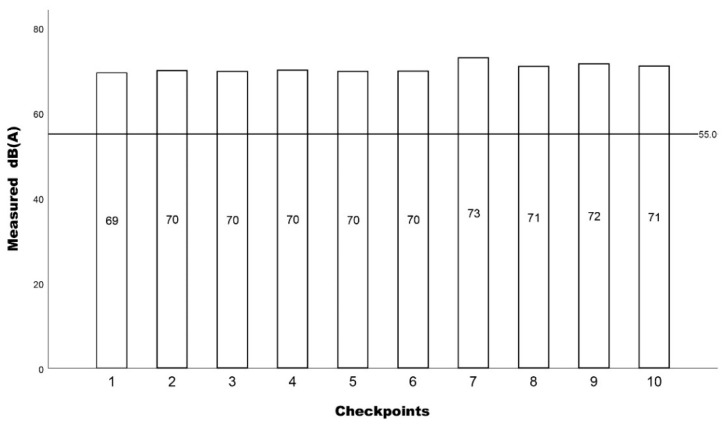
The measured L_eq_ dB(A) results for each checkpoint with a 55 dB(A) reference line.

The results from the measurements conducted are presented in Figure 5. The measured noise levels present slight fluctuations for every recording day, with levels being above the suggested WHO 55 dB(A) levels.

**Figure 6:**
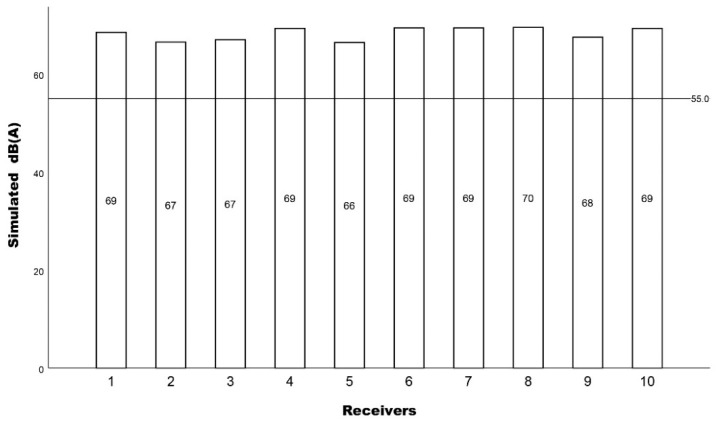
The simulated L_eq_ dB(A) results for each receiver with a 55 dB(A) reference line.

As mentioned, the 11 checkpoints in which the noise level measurements took place were converted into receiver points in order to simulate noise exposure and compare the results with the measured dB(A) levels. The simulation results are presented in Figure 6, and similarly to the measured dB(A) levels, the suggested WHO 55 dB(A) guideline was not met.

**Figure 7:**
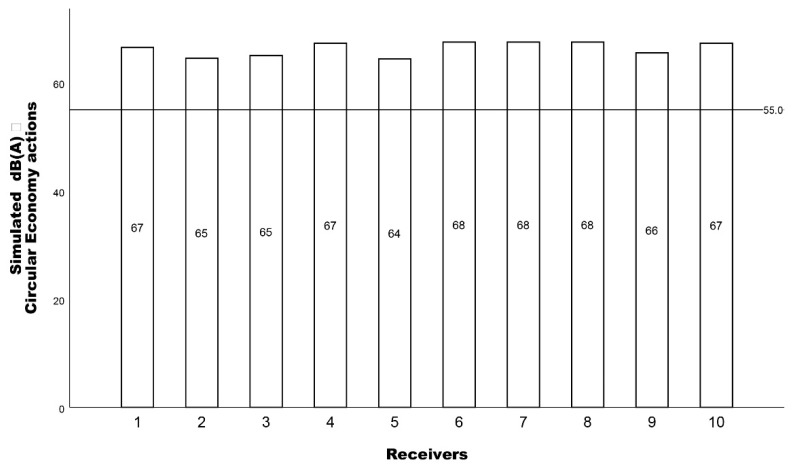
The simulated L_eq_ dB(A) results for each receiver with the circular economy actions incorporated and a 55 dB(A) reference line.

Incorporating circular actions in the simulation procedure, and more specifically, introducing green walls and EVs, the simulation was executed once more. As seen in Figure 7, the dB(A) results resulting from the introduction of circular economy actions still present high noise levels above WHO’s recommendations.

The following step involved the statistical comparison of the measured and the simulated noise levels. According to the Shapiro–Wilk test conducted, the distribution of the data collected was not significantly different from the normal distribution (sig. > 0.05). Therefore, since the assumption of normality had been met, a paired samples *t*-test was conducted.

The descriptive statistics shown in Table 2 provide information regarding the characteristics and distribution of the resulting values, along with the spread and centers of the dataset presented. The resulting skewness and kurtosis levels for the measured noise levels (M = 70.57, SD = 1.066) highlight a right-skewed leptokurtic normal distribution. Additionally, the results regarding the simulated noise levels by the CadnaA noise modeling software (M = 68.38, SD = 1.273) present a left-skewed platykurtic normal distribution. Similarly, the simulated noise levels resulted by the implementation of circular development actions (M = 66.4, SD = 1.289) present a left-skewed platykurtic normal distribution.

**Figure 8:**
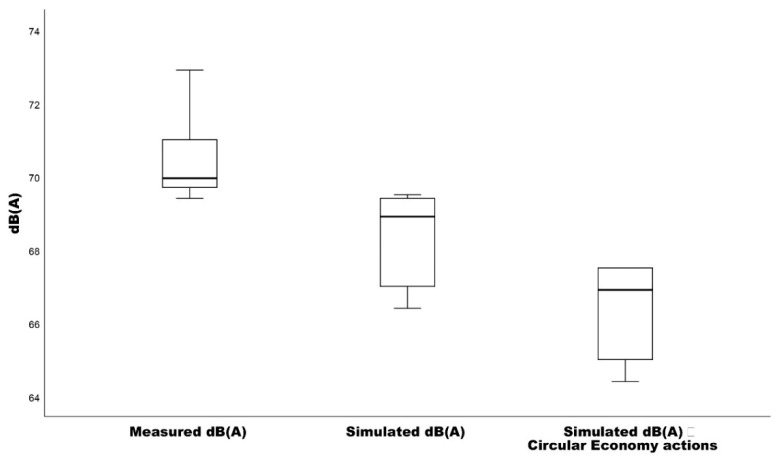
The boxplots showcasing the means of the measured, calculated and calculated with circular economy actions.

The measured and simulated noise levels at the 11 checkpoints/receiver points were statistically compared. A paired samples *t*-test (Table 3) was carried out in order to identify whether there were significant differences among the measured and simulated noise levels. The results for the measured noise levels, in relation to the calculated noise levels with the introduction of circular development actions, differ significantly: t(10) = 5.820, *p* < 0.001, with an effect size of D = 2.700 and a mean difference of 4.17 dB(A), thus rejecting the null hypothesis. Similar results are presented for the two other pairs compared (Figure 8). Furthermore, all resulted effect sizes are very large according to Cohen’s d classification of effect sizes.

**Figure 9:**
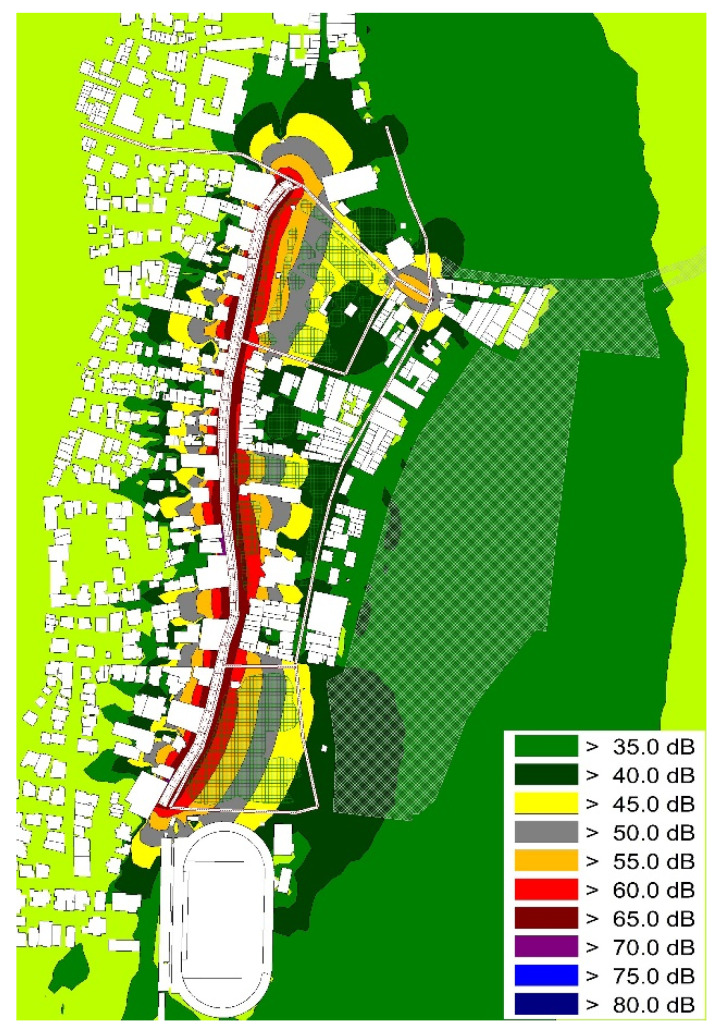
Simulated noise levels. Exposure map utilizing traffic count data without the inclusion of circular economy actions.

**Figure 10:**
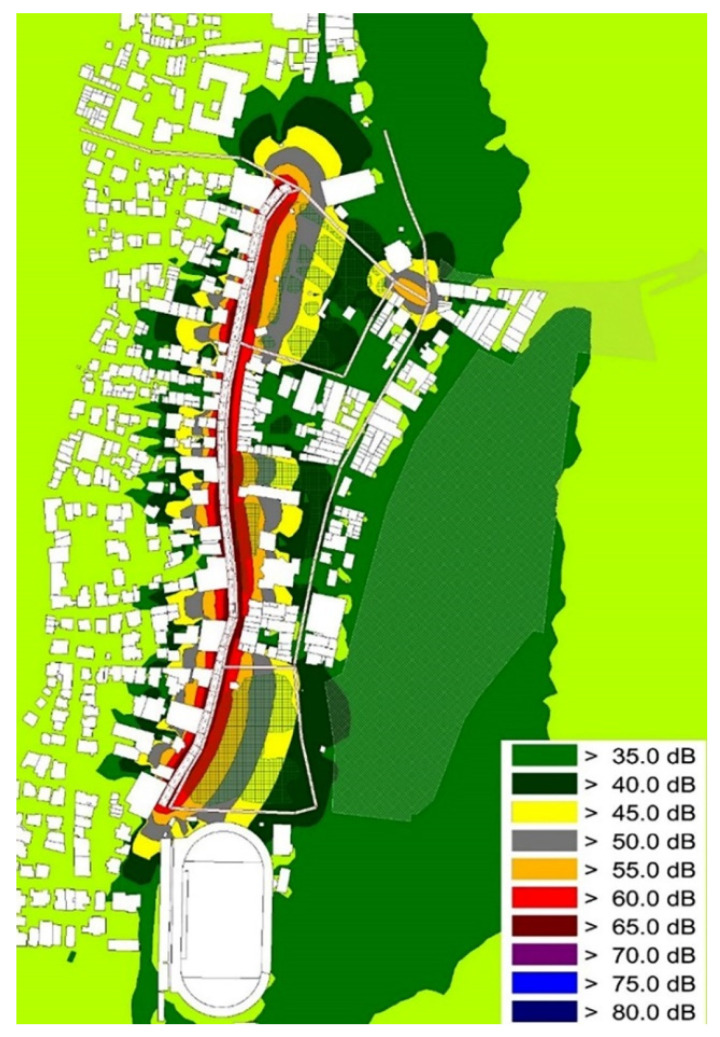
Simulated noise levels. Exposure map utilizing the traffic count data with the inclusion of circular economy actions.

Finally, with the aid of CadnaA Noise Prediction Software, two receiver noise exposure maps were created and are presented in Figure 9 and Figure 10. Despite the existence of statistical differences among the resulted noise levels, there are few visible changes in the noise maps created.

As already met in similar research, the specific noise exposure maps were the result of the traffic counts conducted [142]. In Figure 9, the noise propagation that resulted from road traffic is visualized. Figure 10 presents the noise exposure maps using the same traffic flow data but with the addition of circular actions (green walls and EVs). The two noise exposure maps present a statistical difference of 4 dB(A) at a receiver/checkpoint level.

## 5. Discussion

As already mentioned, the urban circular development actions with an effect on the sound environment of a city able to result in quietness are urban green infrastructure, such as green walls and roofs, and sustainable mobility systems, such as the introduction of electric vehicles. A preliminary modeling experiment was carried out to showcase the possible benefits of green walls and electric vehicles on the sound environment of the small-scale urban area chosen. More specifically, the experiment assessed the effects of the circular actions on the noise levels linked with road traffic, with the aid of the CadnaA noise prediction software.

According to the results (Table 3), a noise level reduction of approximately 4 dB(A) at vehicle speed of 40 km/h for road traffic noise can be expected with the application of two urban circular economy actions (green walls and electro-mobility).

Other experimental studies concerning the absorption coefficient and the noise reduction properties of green walls and green roofs also resulted in a 4 dB(A) noise reduction for traffic noise [106]. Furthermore, it has been documented that the acoustic properties of plants and trees result in noise reduction between 8 and 10 dB(A) in relation to the greenery’s depth and leaf size [143]. Additionally, regarding noise barriers, the noise diffraction properties of greenery in relation to other noise barrier materials has also been calculated.

It is a given fact that electric vehicles (EVs) provide several benefits, including a quieter urban environment [136]. Nevertheless, the specific advantage is strongly correlated with the vehicle’s speed and driving behavior [125]. The propulsion noise reductions found in similar studies differentiate according to the modeling technique and the reference speed used in simulations and measurements, ranging from 10 dB(A) at 10 km/h to 2.5 dB(A) at 50 km/h [144]. Worthy of mention is the introduction of the Acoustic Vehicle Alerting System (AVAS) at speeds below 20 km/h due to the risks involving the low sound levels of EVs and their detectability [145]. Finally, the overall noise reduction has been found to be about 6 dB(A) at 20 km/h but becomes insignificant beyond 40–50 km/h. At low frequencies (below 250 Hz), the EV propulsion noise presents a significant reduction. The global noise reduction of an EV in comparison to an ICEV vehicle at low speed is arbitrarily denoted at −15 dB(A) [125].

Apart from noise mitigation, the studied circular actions present a series of co-benefits: green walls offer a lower indoor temperature; reduced energy usage through better insulation; biodiversity enhancement through the provision of habitats for smaller animals; collection of particulate matter, nitrogen oxides and carbon dioxide; reduced “urban heat island” effect in urban paved areas; and improved microclimate [143]. Similarly, EVs, despite the challenges that need to be addressed in order for them to be considered as fully sustainable, provide a reduction in GHG emissions.

Conventional vehicles occupy mostly the lower frequency bands by emitting low frequency noise. Therefore, EVs may possibly “liberate” a wide frequency range [123,146]. It can be assumed that the release of a frequency occupied by road traffic noise thus far will restore the acoustic niche to its original inhabitants, while enabling the enhancement of biodiversity. It has been proven that cities change the songs of birds constituting them, making them shorter, faster and louder [147], thus requiring more energy to survive. The specific phenomenon—described as the Lombard effect—refers to the noise-dependent regulation of vocal amplitude of animals and humans [148,149] and can result in biodiversity-related implications. Therefore, the introduction of urban circularity actions, apart from the expected noise reduction benefits, will probably result in an overall sound environment alteration and improvement.

### Limitations and Future Work

The current research focused on the effects of road traffic noise on the sound environment of an urban area. Thus, the measurements of noise levels were targeted to minimize other exogenous noise sources. Nevertheless, despite the outcome that green infrastructure elements and developments governed by circularity, such as green walls and electric vehicles, will increase sound absorption and reduce vehicle noise levels, random urban noise events apart from road traffic noise were not taken into consideration.

Further research is needed that will involve more urban sound environment evaluations in the scope of circular economy, including other noise sources, environmental pressures and circular economy actions, in order to reinforce the findings. Furthermore, the implementation of green infrastructure elements such as green walls and roofs and their impact on the structural connectivity of urban green spaces will be put under the research lens.

## 6. Conclusions

Circular urbanism results in several sustainability benefits, including noise mitigation and the creation of urban quietness. This study focused on the incorporation of a circular and bioeconomy approach in noise control efforts, and the relevant long-term sustainability benefits were highlighted.

Using the CNOSSOS-EU noise model guidelines that include traffic counts, vehicle categorizations and noise level measurements, a noise exposure map was created in order to assess the current condition of the sound environment in an urban area of the city of Mytilene, Greece. To assess the benefits that circular economy may provide for the sound environment, e.g., by implementing green wall and electric vehicles, a small-scale simulation was conducted. This simulation resulted in a 4 dB(A) noise reduction in road traffic noise. Similarly to existing related research, speed seems to play an important role in the overall noise exposure levels as it is directly associated with the noise performance of vehicles.

The limitations of this research mainly concern the scale of the area considered and that a simulation of green infrastructure with green walls and electro-mobility facilities was conducted. Nevertheless, a baseline for future comparisons is necessary in the framework of the current program promoting circular interventions in the insular regions of the Aegean (Greece), including the case study island and city.

The results of the simulation prove that the combination of both EVs and greening are promising contributors towards a healthy sound environment. We therefore conclude that implementing a combination of circularity measures enables the mitigation of environmental noise. Strategic urban plans including targets related to acoustic urban sustainability under a bioeconomy and nature-based solutions approach could be conducive towards sustainable urban development and management. However, the challenge today is to move from pilot pioneering projects and experimental work to a real model to build a frugal, resilient and welcoming city with a high-quality sound environment.

## Figures and Tables

**Table 1:** The correction coefficients for ICE vehicles (Kephalopoulos et al., 2012) and for electric vehicles (Pallas et al., 2016).

Octave	Coefficients for Light ICE Vehicles	Correction Coefficients for EVs
125 Hz	85.7 dB	−1.7 dB
250 Hz	84.5 dB	−4.2 dB
500 Hz	90.2 dB	−15 dB
1000 Hz	97.3 dB	−15 dB
2000 Hz	93.9 dB	−15 dB
4000 Hz	84.1 dB	−13.8 dB

**Table 2:** The descriptive statistics of the measured and calculated noise levels.

Descriptive Statistics							
L_eq_ Levels	Mean	SD	Min.	Max.	Skewness	Kurtosis	Variance
Measured dB(A)	70.57	1.066	69.4	72.9	1.041	0.667	1.138
Simulated dB(A)	68.38	1.273	66.4	69.5	−0.690	−1.488	1.622
Simulated Circular Economy dB(A)	66.4	1.289	64.4	67.5	−0.676	−1.503	1.664

**Table 3:** The paired samples *t*-test results and the Cohen’s d effect sizes.

	Paired Samples Test									
	L_eq_ Levels	Mean	SD	Std. Error Mean	95% CI of the Difference		t	df	Sig.	Cohen’s d
					Lower	Upper				
Pair 1	Measured dB(A)	2.19091	1.24856	0.37646	1.35211	3.02971	5.820	10	<0.001	2.700
	Simulated dB(A)									
Pair 2	Measured dB(A)	4.17273	1.25227	0.37757	3.33144	5.01401	11.051	10	<0.001	4.875
	Simulated Circular Economy dB(A)									
Pair 3	Simulated dB(A)	1.98182	0.04445	0.01220	1.95464	2.00899	162.488	10	<0.001	70.124
	Simulated Circular Economy dB(A)									

## Data Availability

Not applicable.
